# Implication of retinoic acid receptor selective signaling in myogenic differentiation

**DOI:** 10.1038/srep18856

**Published:** 2016-02-02

**Authors:** Jihong Chen, Qiao Li

**Affiliations:** 1 Department of Cellular and Molecular Medicine, Faculty of Medicine, University of Ottawa, Ottawa, ON, Canada; 2 Department of Pathology and Laboratory Medicine, Faculty of Medicine, University of Ottawa, Ottawa, ON, Canada

## Abstract

Signaling molecules are important for committing individual cells into tissue-specific lineages during early vertebrate development. Retinoic acid (RA) is an important vertebrate morphogen, in that its concentration gradient is essential for correct patterning of the vertebrate embryo. RA signaling is mediated through the activation of retinoic acid receptors (RARs), which function as ligand-dependent transcription factors. In this study, we examined the molecular mechanisms of RAR-selective signaling in myogenic differentiation. We found that just like natural ligand RA, a RAR-selective ligand is an effective enhancer in the commitment of skeletal muscle lineage at the early stage of myogenic differentiation. Interestingly, the kinetics and molecular basis of the RAR-selective ligand in myogenic differentiation are similar to that of natural ligand RA. Also similar to natural ligand RA, the RAR-selective ligand enhances myogenic differentiation through β-catenin signaling pathway while inhibiting cardiac differentiation. Furthermore, while low concentrations of natural ligand RA or RAR-selective ligand regulate myogenic differentiation through RAR function and coactivator recruitment, high concentrations are critical to the expression of a model RA-responsive gene. Thus our data suggests that RAR-mediated gene regulation may be highly context-dependent, affected by locus-specific interaction or local chromatin environment.

In early vertebrate development, signaling molecules are required to direct individual cells as to when and where to commit to tissue-specific lineages and subsequently differentiate into their designated embryonic tissues. All-*trans* retinoic acid (RA) is one of the vertebrate morphogens playing an important role in patterning and organogenesis^1^,^2^. During early embryogenesis, a RA concentration gradient, spatially regulated by enzymes involved in RA synthesis and degradation, suffuses the embryo and is essential for correct patterning of the vertebrate embryo^3^,^4^.

Mouse embryonic stem (ES) and P19 pluripotent embryonal carcinoma (EC) cells have been employed for many years in developmental biology to delineate molecular mechanisms of stem cell differentiation. These cells respond well to developmental cues *in vitro* to differentiate into cell types of all three germ layers, which simulates the molecular and cellular processes that take place during early embryonic development^5^,^6^. On a molecular level, active RA signaling is found in the neural stem cell niche and pharmacological concentrations of RA (>10^−7^ M) enhance neuronal differentiation of ES and EC stem cells^7^,^8^,^9^. RA also enhances skeletal myogenesis in ES and EC stem cells and in zebrafish embryos^10^,^11^,^12^,^13^,^14^. Particularly, RA has a concentration-dependent effect on the differentiation of ES and EC stem cells, prompting a switch from cardiomyogenesis to skeletal myogenesis at low concentrations (<10^−7^ M), while inhibiting the development of skeletal muscle at high concentrations^15^,^16^.

Skeletal muscle development is a complex process coordinated by multiple myogenic regulatory factors, such as Myf5, MyoD and myogenin^17^,^18^. While Myf5 and MyoD initiate the expression of muscle-specific genes and commit the stem cells into skeletal muscle lineage, myogenin controls terminal differentiation including the fusion of myoblasts to myotubes^19^,^20^,^21^. During RA-enhanced skeletal muscle linage specification, P19 stem cells temporally express a hierarchy of myogenic regulatory factors similar to during embryogenesis. Wnts activated in the primitive streak during mesoderm induction are expressed in the early embryoid bodies (EBs), Pax3 activated in the early somite is subsequently expressed, and then Myf5 and MyoD are expressed marking the commitment and development of skeletal myocytes^14^,^22^.

RA signaling is mediated through the action of retinoic acid receptors (RARs), namely RARα, RARβ and RARγ, which function as ligand-dependent transcription factors. RARs form heterodimers with retinoid X receptors (RXRs: RXRα, RXRβ and RXRγ) and bind constitutively to RA response elements (RAREs) within the regulatory region of target genes^23^. Signaling is initiated following the activation of RARs by RA, which results in the recruitment of the transcriptional coactivator p300 and target gene expression^24^,^25^,^26^. In this doctrine, RXR act as a silent partner since ligand activation is through the binding of RA to RAR. However, natural ligand RA (all-*trans* RA) can be metabolized into 9-*cis* RA that binds and activates not only RAR but also RXR^27^. Hence it is possible that the concentration-dependent effects of natural ligand RA on the differentiation of pluripotent stem cells may stem from a manifold of molecular mechanisms by affecting gene expression through pathways other than RAR selective signaling.

In this study, we investigated the molecular mechanisms of RAR-selective signaling in the commitment of skeletal muscle lineage. We found that the kinetics and molecular basis of a RAR-selective ligand in myogenic differentiation are similar to that of natural ligand RA, suggesting that RA-enhanced myogenic conversion is indeed mediated through the activation of RAR.

## Results

### Effects of RAR selective ligand on myogenic differentiation

In tissue cultures, differentiation of P19 cells can be induced with an EB formation procedure, and EBs formed in the absence of exogenous stimuli express markers of mesoderm, but not of skeletal myocytes^28^. On the other hand, EBs treated with 1% DMSO differentiate into small percentages of skeletal myocytes, and addition of all-*trans* RA at low concentrations enhance myogenic conversion^14^,^22^. However, it is not clear whether this RA effect is mediated solely through the function of ligand-activated RARs, since all-*trans* RA can be metabolized into 9-*cis* RA, and thus RXRs can also be activated. To define the molecular basis of low concentrations of RA in myogenic differentiation, we examined the effects of arotinoid acid, a potent RA analog selective for RARs^29^, on myogenic conversion.

**Figure 1:**
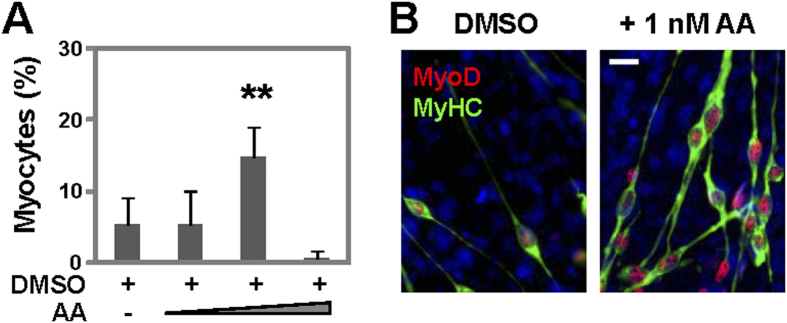
Effects of RAR selective ligand on myogenic conversion. (**A**) Pluripotent P19 cells were treated with increasing amounts of arotinoid acid (AA, 100 pM, 1 nM and 10 nM) and 1% DMSO during EB formation. The cells were then cultured for additional 5 days without any treatments, and stained for microscopic analysis of myosin heavy chain, MyoD and nuclei. Quantification is presented as the percentage of cells differentiated into skeletal myocytes in view of the total cell populations. Error bars represent the standard deviations of three independent experiments. Statistical significance is denoted by ** to indicated p < 0.01 relative to DMSO control. (**B**) Representative microscopic images of myosin heavy chain (MyHC, green), MyoD (red) and nuclei (blue) co-staining (scale bar = 20 μm).

Using the same appraoch as previously reported for RA, we treated P19 pluripotent stem cells with increasing concentrations of arotinoid acid and 1% DMSO during EB formation and maintained the cells for an additional 5 days allowing the development of skeletal myocytes. By day 9 of differentiation, arotinoic acid, at 1 nM (about 30-fold below the Kd), significantly enhanced myogenic conversion when used together with DMSO, generating about 15% skeletal myocytes (Fig. 1A). However, the generation of skeletal myocytes was impaired by the addition of arotinoid acid at a higher concentration, 10 nM (around the Kd, Fig. 1A). This is similar to previous observations that RA, at a low concentration (about 30-fold below the Kd), significantly enhances the differentiation of P19 stem cells into skeletal myocytes, but at a higher concentration (around the Kd) it blocks myogenic differentiation^15^,^16^. Also similar to RA-enhanced myogenic differentiation, MyoD, a marker of terminal specification to the skeletal muscle lineage, co-stained with myosin heavy chain, a muscle structure protein, to the elongated bipolar myoblasts following the addition of arotinoid acid (Fig. 1B).

**Figure 2:**
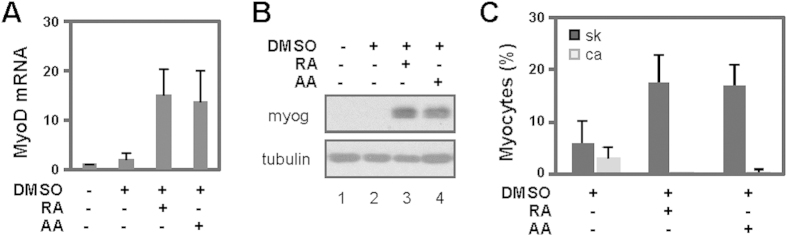
Arotinoid acid inhibits cardiac differentiation of P19 stem cells. (**A**) Cells were differentiated with RA (10 nM) or arotinoid acid (AA, 1 nM) in the presence of DMSO during EB formation and cultured without any treatments. Quantitative PCR was used to determine the mRNA levels of MyoD on day 9 of differentiation, and presented as the fold change in reference to untreated cells normalized to GAPDH (n = 3). (**B**) Protein levels of myogenin were determined using Western. The blots were then stripped and reprobed for β-tubulin as loading controls. Shown are the cropped blot images representing indicated proteins. (**C**) The cells were stained for microscopic analysis in parallel. Quantification of differentiation is presented as the percentage of skeletal myocytes (sk, dark grey) or cardiomyocytes (ca, light grey) in relation to the total cell populations (n = 5).

More importantly, the mRNA of MyoD was augmented following the addition of arotinoid acid to a similar degree as with RA, correlating with their efficacies on the specification of skeletal muscle lineage (Fig. 2A). Furthermore, myogenin, an identity marker of skeletal myocytes was detected using Western analysis by day 9 of differentiation following the addition of arotinoid acid and was comparable in a degree to cells treated by RA (Fig. 2B). The detection of myogenin protein as a measure of myogenic differentiation provides a definite determination of the presence of skeletal myocytes, since myogenin expression is integral to the development of skeletal myocytes. Thus, arotinoid acid, just like RA, is an effective enhancer in the specification and the commitment of the muscle lineage at the early stage of myogenic differentiation, underscoring the importance of RARs in the development of skeletal myocytes.

### Inhibition of cardiac differentiation by arotinoid acid

In addition to differentiating into skeletal myocytes, pluripotent P19 stem cells treated with DMSO can also differentiate into small percentages of cardiomyocytes^11^,^30^. Consistent with previous reports, following 1% DMSO treatment, P19 cells produced about 3% of cardiac and 6% of skeletal myocytes as determined by quantitative immunofluorescence microscopy (Fig. 2C). More interestingly, while the low concentration of arotinoid acid enhanced the specification of skeletal muscle lineage by about 3-fold, it blocked the production of cardiomyocytes (Fig. 2C). In parallel, the addition of RA also attenuated cardiac differentiation while enhancing the generation of skeletal myocytes (Fig. 2C), which is consistent with previous observations of an inhibitory effect of RA on cardiac differentiation^14^,^30^. Taken together, our data suggests that the effects of low concentration of RA on cardiac and skeletal differentiation, just like arotinoid acid, may be mediated through the activation of RARs.

### Roles of β-catenin and RAR in arotinoid acid-enhanced myogenic conversion

**Figure 3:**
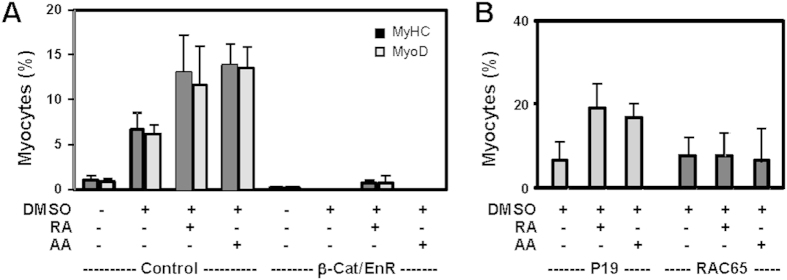
Role of β-catenin and RAR in arotinoid acid-enhanced myogenic conversion. (**A**) A line of P19 stem cells expressing a dominant negative β-catenin (β-cat/EnR) was differentiated with RA (10 nM) or arotinoid acid (AA, 1 nM) in the presence of DMSO. The cells were then maintained on coverslips without any treatments for an additional 5 days and stained with antibodies against MyoD, myosin heavy chain and with Hoechst for nuclei. Cells harbouring the empty vector were used as controls. Quantification is plotted as the percentage of cells differentiated into skeletal myocytes relative to the total cell populations. Error bars represent the standard deviations of three independent experiments. (**B**) RAC65 cells were differentiated with RA or arotinoid acid in the presence of DMSO and analyzed for the expression of myosin heavy chain using quantitative immunofluorescence microscopy. The parental P19 cells were used as controls. Quantification is plotted as the percentage of cells differentiated into skeletal myocytes relative to the total cell populations. Error bars represent the standard deviations of four independent experiments.

**Figure 4:**
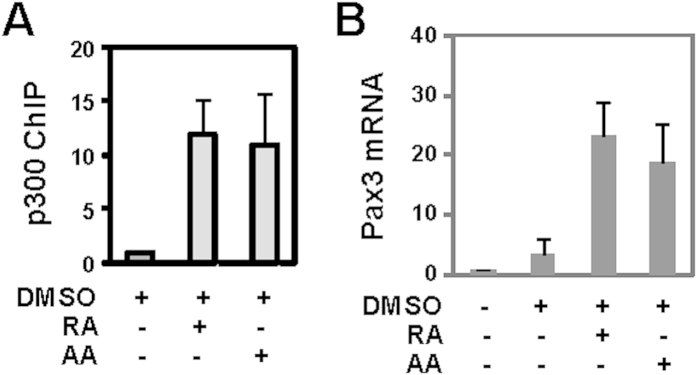
Enrichments of transcriptional coactivator p300 at the Pax3 locus. (**A**) Cells were differentiated with RA (10 nM) or arotinoid acid (AA, 1 nM) in the presence of 1% DMSO during EB formation and qChIP was used to examine enrichment of p300 at the Pax3 locus on day 4 of differentiation. Quantification is presented as the fold change in reference to DMSO control. Error bars represent the standard deviations of four independent experiments. Input DNA was used as internal controls. (**B**) Real-time PCR was used to determine the mRNA levels of Pax3 in parallel, and presented as the fold changes in reference to untreated EBs normalized to GAPDH (n = 3).

To delineate the molecular pathway of arotinoid acid in myogenic differentiation, we next took advantage of a line of established P19 stable cells, since RA-enhanced skeletal myogenesis depends on the function of β-catenin^14^,^22^. To determine the requirement of β-catenin for arotinoic acid-enhanced myogenesis, we employed cells stably expressing a dominant negative β-catenin in which the transcriptional activation domain is replaced by an engrailed repressor domain^14^, which silences gene transcription by interacting with the members of Groucho/TLE family of transcriptional repressors^31^. Cells harbouring the empty vector were used in parallel as a control. As shown in Fig. 3A, the control cells differentiated into skeletal myocytes following the addition of RA or arotinoid acid, to comparable extents as the parental cells (compare with Fig. 2C). However, the dominant negative β-catenin cells failed to differentiate into skeletal myocytes regardless of treatment (Fig. 4A). Therefore, arotinoic acid acts through the same molecular pathway as RA and cannot bypass β-catenin to direct skeletal myogenesis, and the function of β-catenin is essential for arotinoid acid-enhanced myogenic conversion.

To further delineate whether RAR is important for arotinoid acid-enhanced skeletal myogenesis, we employed a line of RAC65 cells that contain a dominant negative RARα, which effectively blocks the DNA binding capacity of the receptors^32^,^33^. These cells are non-responsive to RAR agonist, but respond to RXR agonist to undergo neuronal and myogenic differentiation^22^,^34^,^35^. As shown in Fig. 3B, again just as RA, arotinoid acid was not able to enhance myogenic conversion in RAC65 cells. Thus, RA directs the specification of skeletal muscle through the action of RAR and through molecular pathways similar to arotinoid acid.

### p300-dependent arotinoid acid signaling in myogenic conversion

A RA responsive region has been previously identified at a Pax3 locus^14^,^22^. Particularly, the occupancy of transcriptional coactivator p300 at this region is significantly augmented in RA-enhanced myogenic conversion^22^. We thus examined the effect of arotinoid acid on p300 enrichment at this Pax3 locus. Quantitative ChIP analysis showed that the occupancy of the transcriptional coactivator p300 to this region was augmented by the addition of arotinoid acid to a similar degree as with RA (Fig. 4A). In addition, levels of Pax3 mRNA were also increased to a similar degree following treatments with arotinoid acid and RA (Fig. 4B). Therefore, arotinoid acid enhances myogenic differentiation at least in part through the activation of muscle progenitor factor Pax3, which is a well-characterized gene target for RA action at low concentrations^14^,^22^.

### Effects of arotinoid acid on RA-responsive gene activation

**Figure 5:**
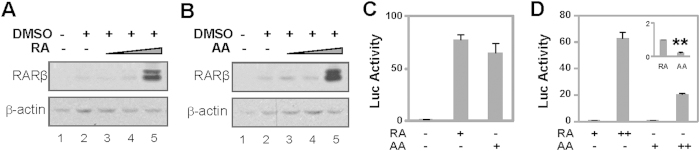
RAR agonist responsive transcriptional activation. (**A**) P19 stem cells were treated with increasing concentrations of RA (100 pM, 10 nM, 1 μM) in the presence of DMSO during EB formation. Protein levels of RARβ were analyzed using Western on day 4 differentiation. The blots were then stripped and reprobed for β-actin as loading controls. Shown are the cropped blot images representing indicated proteins. (**B**) Cells were treated with increasing concentrations of arotinoid acid (AA, 100 pM, 1 nM and 10 nM) in the presence of DMSO during EB formation and analyzed for the levels of RARβ protein on day 4 of differentiation. The blots were then stripped and reprobed for β-actin as loading controls. Shown are the cropped blot images representing indicated proteins. (**C**) P19 stem cells were transfected with a RARE luciferase reporter (0.1 μg) and a RSV-β-Gal (0.2 μg), treated with RA (10 nM) or arotinoid acid (AA, 1 nM) for 24 hours and harvest for luciferase assay. Values are the fold induction of RA or arotinoic acid compared to untreated control. β-Gal activity was used as internal controls. (**D**) Values of the luciferase activity are the fold induction of RA or arotinoid acid at the high concentration compared with of RA at the low concentration (10 nM). Inset is the transcriptional activity of arotinoid acid in fold change relative to that of RA (**p < 0.01, n = 3).

To further determine the effects of arotinoid acid on RA-responsive gene activation, we first examined the expression of endogenous RARβ2 gene during EB formation, since this gene contains a consensus RARE and its expression is rapidly induced by RA^36^,^37^. As shown in Fig. 5A,B, a detectable level of RARβ protein was found using Western blotting following DMSO treatment as previously reported^38^. However, only the high concentrations, but not the low concentrations of RA or arotinoid acid, were able to markedly enhance RARβ gene expression (Fig. 5A,B).

We next used a luciferase reporter containing the RARE segment of the RARβ2 promoter to examine the effects of arotinoid acid on transactivation through the action of RAR. The cells were transfected with this well characterized RA-responsive reporter^39^, treated with RA or arotinoid acid, and then harvested for the luciferase assays. Consistent with previous reports, RA and arotinoid acid were able to induce transcriptional activation of this reporter (Fig. 5C,D). In particular, treatment with low concentrations of RA and arotinoid acid induced the activity of luciferase by about 70-fold, in comparison to untreated control (Fig. 5C) and high concentrations of RA and arotinoid acid further induced the activity of luciferase by another 20- and 60-fold, respectively, compared to the low concentration of RA (Fig. 5D). Interestingly, the high concentration of RA had a more robust transcriptional activity (Fig. 5D), possibly due to RA metabolism. Taken together, our data suggests that while high concentrations of RA and arotinoid acid (around the kd) act through classical activation of RAR as a transcriptional regulator, low concentrations of RA and arotinoid acid (30-fold below the Kd) may act through context-dependent RAR activation to commit stem cells into the skeletal muscle lineage (Figs 4 and 5).

## Discussion

We have examined the kinetics and molecular action of arotinoid acid, a RAR-selective ligand, in myogenic conversion as compared to natural ligand RA. Our findings show that similar to RA, arotinoid acid enhances commitment to the skeletal muscle lineage at a narrow concentration range (about 30-fold below the Kd). Also similar to natural ligand RA, the RAR-selective ligand enhances myogenic differentiation through the β-catenin pathway, while inhibiting cardiac differentiation. Moreover, similar to the natural ligand RA, arotinoid acid enhances myogenic conversion in a RAR-dependent manner. Thus our data suggests that RAR-mediated gene regulation may be highly context-dependent, affected by locus-specific signature or local chromatin environment.

RA signaling is essential for normal embryonic development and growth, thus deficiency in RA signaling during early embryogenesis leads to congenital malformations affecting patterning and the development of many organ systems^3^,^40^. While RXRα/RAR heterodimers are the main functional units mediating RA signals, the pleiotropic effects of RA on development reflect complex combinatorial mechanisms, through which the RXR/RAR heterodimers are differentially activated to selectively control the expression of different RA target genes^1^,^41^. Particularly regarding RXR within the RXR/RAR heterodimer, its transcriptional activity can be subordinated to ligand activation of the RAR thus acting as an silent partner, or can be primary following activation by its own ligand thus acting in synergy with the RAR, depending on the nature of the RA-controlled developmental event^1^. It is well established that the efficiency of cellular differentiation is influenced by the concentration of RA^7^,^34^. The working concentration of RA for myogenic differentiation is well below the Kd^14^,^22^. Since natural ligand RA can be metabolized into different metabolites that can activate the RXR, and RXR selective ligand has the capacity to augment myogenic differentiation^22^, the question remains if RA-enhanced myogenic differentiation is mediated through a RAR-selective mechanism.

Here, we show that arotinoid acid, a RAR-selective ligand, just like natural ligand RA is an effective enhancer to commit stem cells to skeletal muscle lineage at the early stage of myogenic differentiation (Figs 1 and 2). We also show that the functions of β-catenin and RAR are essential for arotinoid acid-enhanced myogenic conversion similar to RA (Fig. 3). Taken together, our data demonstrate that natural ligand RA directs the development of skeletal muscle through molecular pathways in a similar manner as arotinoid acid, underscoring the activation of RARs during this process, albeit at concentrations below the Kd.

Like other nuclear receptors, RARs are known as a class of transcription factors that are able to initiate dynamic chromatin changes in RA-responsive loci through recruiting chromatin modifying enzymes and p300^42^,^43^,^44^,^45^,^46^,^47^,^48^,^49^. While the low concentration of RA regulates myogenic differentiation through the action of RAR, it exhibits differential efficacy in the expression of a model RA responsive gene (Fig. 5). This may due to the composition of RARE possibly similar to the mechanisms of combinatorial gene regulation by glucocorticoid receptor (GR) in which hormone induced transcriptional response correlates with GR binding site affinity that is determined by GRE architecture and co-regulator expression^50^. Taken together, our data suggests that while high concentrations of RA and arotinoid acid (around the kd) acts through classic activation of RAR as a transcriptional regulator, low concentrations of RA and arotinoid acid (30-fold below the Kd) may act through context-dependent RAR activation and concerted action with muscle related regulators to commit stem cells to the skeletal muscle lineage (Figs 4 and 5). Understanding the molecular mechanisms of these interactions will be critical to comprehend at a molecular level how different signaling pathways and chromatin modifying activities converge to regulate cellular differentiation.

## Methods

### Cell culture and reagents

P19 pluripotent stem cells (ATCC) were maintained in Dulbecco’s Modified Eagle Medium (DMEM) containing 5% fetal bovine serum and 5% bovine calf serum at 37 °C and 5% CO_2_. To induce myogenic differentiation, cells were first grown in Petri dishes for 4 days to allow the development of EBs under the indicated treatment conditions and then plated onto coverslips coated with 0.1% gelatin or tissue culture dishes for an additional 5 days without treatment to develop skeletal myocytes^51^. All-*trans* RA and arotinoid acid (4-[(E)-2-(5,6,7,8-Tetrahydro-5,5,8,8-tetramethyl-2-naphthalenyl)-1-propenyl]benzoic acid) were dissolved in ethanol and stored at −20 °C. Dimethyl sulfoxide (DMSO) was stored at room temperature. All chemicals were from Sigma-Aldrich.

### Immunofluorescence microscopy

Following myogenic conversion, the cells were washed with phosphate buffered saline (PBS), fixed in methanol, rehydrated with PBS and incubated with primary antibodies for myosin heavy chain and MyoD at 4 °C overnight. The cells were washed with PBS and incubated with Alexa Flor^®^488 and Flor^®^594 secondary antibodies (Invitrogen) at room temperature for 45 minutes. The cells were also incubated with Hoechst (0.5 mg/ml, Molecular Probes) for 5 minutes for DNA staining. The coverslips were mounted onto the slides in 50% glycerol^52^. Immunoflorescence images were captured with an Axiovert 200M microscope (Zeiss), AxioCam HRM camera (Zeiss) and AxioVision Rel 4.6 software (Zeiss) through different filters^53^. For each coverslip, about 100 fields of view were analyzed with at least 200 cells in each field of view. The efficiency of skeletal myogenic differentiation was estimated based on elongated bipolar cells positively stained for myosin heavy chain in relation to the total cell populations as determined by Hoechst staining, whereas estimation of cardiac differentiation was based on rounded myosin heavy chain positive cells^22^,^30^,^54^. Student *t*-tests were used for statistical analysis. Antibody for myosin heavy chain was from MF20 hybridoma and for MyoD was from Santa Cruz Biotechnology.

### Western analysis

Following myogenic conversion, the cells were washed with PBS and centrifuged at 3,000 *g* for 3 minutes at 4 °C. The cell pellets were suspended with whole-cell extract buffer containing 50 mM Tris-HCl (pH 7.6), 400 mM NaCl, 10% glycerol, 1 mM dithiothreitol, 1 mM phenylmethylsulfonyl fluoride and 1% nonidet P-40 (NP-40), and incubated at 4 °C for 30 minutes. Following incubation, cell lysates were centrifuged at 14,000 *g* for 20 minutes at 4 °C. The protein concentrations of the supernatants were determined using the Bradford assay (Bio-Rad). Western blotting was performed using myogenin antibody detected with Western Lightning™ Chemiluminescence reagents^55^. Myogenin and β-tubulin antibodies were from F5D and E7 hybridomas, respectively.

### Real-Time PCR analysis

The total cellular RNA was isolated using a Total RNA Kit I according to the manufacture’s recommendations (Omega), and reverse-transcribed using a high-capacity cDNA Reverse Transcription Kit (Applied Biosystems) as described previously^56^. Real-time PCR was performed using the SYBR Green and ROX PCR Master Mix HotStarTaq DNA polymerase kit (Qiagen) with the Applied Biosystems 7500 Fast real-time PCR system. The amount of gene targets, normalized to the GAPDH endogenous reference and relative to a calibrator control, was calculated using the arithmetic formula 2^−ΔΔCT^.

### ChIP analysis

Following EB formation, the cells were fixed with formaldehyde on day 4 of differentiation, sonicated with a Bioruptor^®^ and the chromatin was immunoprecipitated with a p300 antibody as described previously^22^,^57^. A sample of 5% of total chromatin was used as the input control. The antibody against p300 was from Santa Cruz Biotechnology and the negative control IgG antiserum was from Zymed Laboratories Inc. The immunoprecipitated DNA was purified using the Omega Bio-tek Cycle Pure Kit, and the purified DNA samples were analyzed using SYBR Green Real-Time PCR. Primer pairs used for PCR amplification were described previously^14^.

### Luciferase assay

Transfection was performed with the reporter plasmids by using ExGen 500 as described previously^52^. Briefly, cells were transfected with a RAR luciferase and RSV-β-Gal reporter, and then treated with RA or arotinoid acid for 24 hours. Luciferase assays were performed according to the manufacturer’s recommendations (Promega). The luciferase activities are expressed as the fold induction in relation to the indicated controls after being normalized to β-galactosidase activity.

## Additional Information

**How to cite this article**: Chen, J. and Li, Q. Implication of retinoic acid receptor selective signaling in myogenic differentiation. *Sci. Rep.* **6**, 18856; doi: 10.1038/srep18856 (2016).
